# Determination of reference intervals for metabolic profile of Hanwoo cows at early, middle and late gestation periods

**DOI:** 10.1186/s40104-015-0009-0

**Published:** 2015-03-18

**Authors:** Da Chuan Piao, Tao Wang, Jae Sung Lee, Renato SA Vega, Sang Ki Kang, Yun Jaie Choi, Hong Gu Lee

**Affiliations:** Laboratory of Animal Cell Biotechnology, Department of Agricultural Biotechnology, Seoul National University, Shinlim-dong, Kwanak-gu Seoul, 151-742 South Korea; College of Animal Science and Technology, Jilin Agricultural University, 2888 Xincheng Street, Nan-guan District Changchun, 130118 People’s Republic of China; Key Laboratory of Animal Nutrition and Feed Science, Jilin Province, Jilin Agricultural University, 2888 Xincheng Street, Nan-guan District Changchun, 130118 People’ Republic of China; Department of Animal Science and Technology, College of Animal Bioscience & Technology, Konkuk University, 120 Neungdong-ro, Gwangjin-gu Seoul, 143-701 South Korea; Animal & Dairy Sciences Cluster, College of Agriculture, University of the Philippines Los Bañose, Los Baños, 4031 Laguna Philippines

**Keywords:** Hanwoo cows, Metabolic profile, Reference intervals, Wagyu cows

## Abstract

**Background:**

Metabolic profile was initially designed as a presymptomatic diagnostic aid based on statistical analyses of blood metabolites to provide an early warning of certain types of metabolic disorder. However, there is little metabolic profile data available about Korean Hanwoo cows. Therefore, this study aimed to determine the reference intervals of metabolic profile for Korean Hanwoo cows.

**Methods:**

Healthy animals (2,205) were selected and divided into early (day 1 to 95), middle (day 96 to 190) and late (day 191 to 285) period according to their gestating period. Metabolic profile including total protein (TP), albumin (Alb), urea (UREA), glucose (Glu), total cholesterol (T-Cho), long-chain fatty acid (LCFA), aspartate aminotransferase (AST), gamma-glutamyl transpeptidase (GGT), creatinine (Crea), calcium (Ca), inorganic phosphorous (iP) and magnesium (Mg) were analyzed using a TBA-40FR automatic biochemical analyzer. The data of Korean Hanwoo cows were then compared to those of the Japanese Wagyu cows.

**Results:**

Most of the data of the Korean Hanwoo cows were relatively higher than those of Japanese Wagyu cows, with the exception of Glu and GGT. This may indicate that the nutritional level of feed for the Korean Hanwoo cows was higher than that of the Japanese Wagyu cows because of the different feeding system. In particular, relatively higher levels of UREA and LCFA were observed in the Korean Hanwoo cows, and this may also contribute to the low reproduction efficiency.

**Conclusions:**

These findings may provide some theoretical basis for understanding the reproductive and feeding situation of Korean Hanwoo cows.

## Introduction

Blood metabolites reflect the nutritional status as well as the physiological condition of an animal. The physiology of a cow changes in the peripartum period, and it is important to monitor nutritional and physiological status rapidly and precisely, because these cows are prone to peripartum metabolic disorders and reproductive diseases. The metabolic profile was initially designed as a presymptomatic diagnostic aid based on statistical analyses of blood metabolites to provide an early warning for certain types of metabolic disorders [1]. Subsequently, metabolic profile has been applied to assess nutritional status [2,3], improve feeding management and diagnose metabolic disorders in dairy herds [4,5]. Reference intervals are useful when interpreting a set of metabolic profile results. Payne et al. [6] estimated normal intervals (95% confident interval) using 2,400 blood samples from 13 dairy herds. Kida [4] established a 10-day criteria for metabolic profile by using data from 29,043 cows in 1,130 commercial dairy herds covering dry and lactation periods [5]. The practicability of the criteria was evaluated in herds with peripartum diseases, and the metabolic abnormalities were successfully detected not only in the herd but also in individual cows. However, little metabolic profile data has been found about Korean Hanwoo cows. Therefore, in the current study, twelve blood metabolites were analyzed in order to determine reference intervals of metabolic profile at the early, middle and late reproduction period in Korean Hanwoo cows. Moreover, the data of Korean Hanwoo cows were also compared to those of Japanese Wagyu cows obtained from NOSAI [7].

## Materials and methods

### Experiment design, animals and sampling

A total of 2,205 healthy Korean Hanwoo cows were selected from various farms in Jangsu-gun Jeollabuk-do, South Korea from 2006 to 2011. All experimental procedures were in accordance with the “Guidelines for the Care and Use of Experimental Animals of Seoul National University”. No abnormalities in these animals were observed or monitored. Generally, all cows were housed indoors and some were on pasture in summer. Feeding systems included continuous feeding of a total mixed ration or separate feeding of forage and concentrates. The metabolic profile was conducted through four seasons. The animals were sorted into four groups according to the plasma pregnancy-associated glycoproteins (PAGs) (Table 1). Blood samples were taken via the external jugular vein after the morning meal. Serum was recovered from the blood samples through centrifugation at 3,500 rpm at 4°C for 15 min, and stored at −80°C until required. All samples were analyzed within one week of being collected.

**Table 1:** **Classification of cows into four stages according to pregnant status**

**Reproduction period**	**Duration**
Non-pregnant	After calving or before pregnancy
Early-pregnant^1^	Day 1 to 95
Middle-pregnant	Day 96 to 190
Late-pregnant	Day 191 to 285

^1^The pregnancy tests were defined based to the plasma pregnancy-associated glycoproteins (PAGs) levels.

### Analysis of metabolic profile

The metabolic profile was performed with a Toshiba Accute Biochemical Analyzer-TBA- 40FR (Toshiba Medical Instruments, Otawara-shi, Tochigi-ken, Japan) according to a previously described method [5]. Indicators for protein metabolism consist of total protein (TP), albumin (Alb) and urea (UREA); for energy metabolism, glucose (Glu), total cholesterol (T-Cho) and long-chain fatty acids (LCFA); for liver and kidney function, aspartate aminotransferase (AST) and γ-glutamyl transpeptidase (GGT) and Creatinine (Crea); for mineral metabolism, calcium (Ca), inorganic phosphorus (iP) and magnesium (Mg) (Table 2). All the reagents required for this procedure were purchased from Wako Pure Chemical Industries, Ltd. (Chuo-ku, Osaka, Japan). The reference intervals were determined by following the recommendations of the Clinical and Laboratory Standards Institute (CLSI).

**Table 2:** **Analytical method used for metabolic profile**

**Components**	**Method of analysis**
*Protein metabolism*	
Total protein (TP)	Biuret Test
Albumin (Alb)	Bromcresol green (BCG) Method
Urea (UREA)	Urease-Glutamate dehydrogenase (GLDH) Method
*Energy metabolism*	
Glucose (Glu)	Hexokinase (HK)-
Glucose-6-phosphate dehydrogenase (G6PD) Method	
Total cholesterol (T-Cho)	Cholesterol oxidase
Long-chain fatty acid (LCFA)	Enzymatic colorimetric
*Liver function*	
Aspartate aminotransferase (AST)	Malate dehydrogenase (MDH) UV
Gamma glutamyl transpeptidase (GGT)	Glu-3-CA-4-NA substrate
*Kidney function*	
Creatinine (Crea)	Jaffe’ Method
*Mineral metabolism*	
Calcium (Ca)	O-Cresolphthalein Complexone (OCPC) Method
Inorganic phosphorous (iP)	Enzymatic UV
Magnesium (Mg)	Enzymatic UV

### Statistical analysis

The metabolic profile data were presented as Mean ± SD and analyzed using a one-way analysis of variance (one-way ANOVA) (SPSS Inc., Chicago, IL, USA). In all cases, differences were considered significant if *P* < 0.05.

## Results and discussion

The metabolic profile of Korean Hanwoo cows in different physiological stages were determined according to data collected from 2,205 animals (Table 3). Protein metabolism: It has been well known that TP, Alb and UREA levels are indicators of sufficient protein intake from diets. Alb is not a long-term indicator of protein intake because of its relatively short half-life in the blood. On the other hand, UREA level is a good indicator of long-term intake of dietary protein [8]. TP, Alb and UREA were significantly (*P* < 0.05) increased from the EP to LP periods, suggesting that there was an increased intake of diet. The mean level of TP was slightly higher than in previous reported studies [9]. Energy metabolism: In the past three decades, the question of whether Glu can be used as indicator for energy metabolism has been discussed [8]. Blood Glu has a moderate diagnostic value in the assessment of nutritional status of cattle, as it varies moderately in blood [10]. It has also been reported that glucose can be used in combination with other indicators to assess energy metabolism [9]. The serum Glu concentration detected in this study was similar with one previously reported [9], but lower than some other reported studies in other species [11,12]. These differences may be attributed to the species differences or the feeding system. Significantly higher levels of LCFA were observed at the EP and LP stage (*P* < 0.05). Liver function and kidney function: AST and GGT are enzymes that indicate liver cell damage and biliary obstruction, respectively. In this study, the mean level of AST was higher than in previously reported studies [9]. Therefore, it seemed that the high level of AST reveals early signs of liver cell damage in Korean Hanwoo cows. Mineral metabolism: Serum concentrations of Ca and IP reflect dietary calcium and phosphate intake [8], and it is well known that serum Ca is under homeostatic control of the endocrine system. Ca, IP and Mg have a high diagnostic value in determining the nutritional status of animals due to their low variability in blood [10]. In this study, low variability of the three indicators was also observed, but the mean values of these three indicators were higher than in previously reported studies. This may be due to the high level of mineral supplementation in the diet of Korean Hanwoo cows.

**Table 3:** **Serum metabolic profile of Korean Hanwoo cows at different stages of pregnancy**

**Indicator**	**NP** ^**1**^		**EP** ^**1**^		**MP** ^**1**^		**LP** ^**1**^	
	**Mean**	**SD**	**Mean**	**SD**	**Mean**	**SD**	**Mean**	**SD**
TP, g/dL	7.56^a^	0.82	7.61^ab^	0.83	7.73^c^	0.92	7.69^b^	0.79
Alb, g/dL	3.60^a^	0.31	3.62^ab^	0.29	3.65^b^	0.31	3.71^c^	0.30
UREA, mg/dL	10.40^a^	3.50	11.10^b^	3.50	11.40^b^	3.20	11.50^b^	4.10
Glu, mg/dL	39.00^a^	19.00	39.00^a^	18.00	41.00^a^	18.00	45.00^b^	18.00
T-Cho, mg/dL	128.00	36.00	130.00	35.00	126.00	33.00	131.00	36.00
LCFA, μEq/L	175.00^b^	145.00	137.00^a^	106.00	163.00^b^	108.00	225.00^b^	193.00
AST, IU/L	75.00^b^	16.00	74.00^ab^	17.00	72.00^a^	19.00	74.00^ab^	18.00
GGT, IU/L	17.00^a^	6.00	18.00^b^	7.00	17.00^a^	6.00	17.00^a^	6.00
Crea, mg/dL	1.43^a^	0.37	1.46^a^	0.36	1.55^b^	0.35	1.56^c^	0.44
Ca, mg/dL	10.24^bc^	0.87	10.14^b^	0.87	10.14^a^	1.09	10.30^c^	1.14
IP, mg/dL	6.75	1.88	6.57	2.15	6.79	1.63	6.68	2.96
Mg, mg/dL	2.57^a^	0.35	2.60^a^	0.37	2.59^a^	0.38	2.67^b^	0.45

^1^NP: non-pregnant, n = 592; EP: early pregnant, n = 686; MP: middle pregnant, n = 517; LP: late pregnant, n = 410.

^a-c^Values followed by different letters within each component are significantly different (***P*** < 0.05).

When interpreting laboratory data for metabolic profile from a herd or individual animal, reference intervals need to be determined for clinical application. In this experiment, we also determined reference intervals for serum components at 4 physiological stages in Korean Hanwoo cows, as are shown in Table 4. It was found that reference intervals were changed according to physiological status. These reference intervals may provide a basis for interpreting data analysis and metabolic disorder. A comparison between Korean Hanwoo and Japanese Wagyu cows was performed. The amounts of serum components are affected by several factors including nutrition, physiological status, breed, season and age.

**Table 4:** **Reference intervals of serum components of Korean Hanwoo cows at different stages of pregnancy**

**Indicator**	**NP** ^**1**^		**EP** ^**1**^		**MP** ^**1**^		**LP** ^**1**^	
	**D** ^**2**^	**Intervals**	**D**	**Intervals**	**D**	**Intervals**	**D**	**Intervals**
TP, g/dL	G^2^	5.9 ~ 9.2	N	6.4 ~ 9.6	N	6.5 ~ 10.4	N	6.4 ~ 9.8
Alb, g/dL	G	2.97 ~ 4.22	G	3.04 ~ 4.20	G	3.03 ~ 4.27	G	3.11 ~ 4.31
UREA, mg/dL	G	3.4 ~ 17.4	G	4.0 ~ 18.1	G	4.9 ~ 17.8	N	4.7 ~ 17.9
Glu, mg/dL	N^2^	9 ~ 57	N	7 ~ 57	N	7 ~ 57	N	4 ~ 69
T-Cho, mg/dL	G	56 ~ 200	G	60 ~ 200	G	60 ~ 192	G	59 ~ 203
LCFA, μEq/L	N	37 ~ 525	N	30 ~ 430	N	35 ~ 417	N	45 ~ 699
AST, IU/L	G	43 ~ 107	N	46 ~ 113	N	49 ~ 116	N	48 ~ 126
GGT, IU/L	N	7 ~ 32	N	6 ~ 38	G	5 ~ 29	N	4 ~ 31
Crea, mg/dL	G	0.69 ~ 2.17	N	0.74 ~ 2.18	G	0.85 ~ 2.25	G	0.68 ~ 2.0
Ca, mg/dL	G	8.5 ~ 11.9	N	8.5 ~ 11.5	N	9.0 ~ 11.8	G	8.9 ~ 12.5
IP, mg/dL	G	3.1 ~ 10.5	N	3.2 ~ 10.8	G	3.5 ~ 10.5	N	3.6 ~ 10.2
Mg, mg/dL	G	1.87 ~ 3.27	G	2.23 ~ 3.34	G	1.80 ~ 3.35	N	2.04 ~ 3.27

^1^NP: non-pregnancy, n = 592; EP: early pregnancy, n = 686; MP: middle pregnancy, n = 517; LP: late pregnancy, n = 410.

^2^D: Distribution of data; G: Gaussian distribution; N: Non-Gaussian distribution.

**Figure 1:**
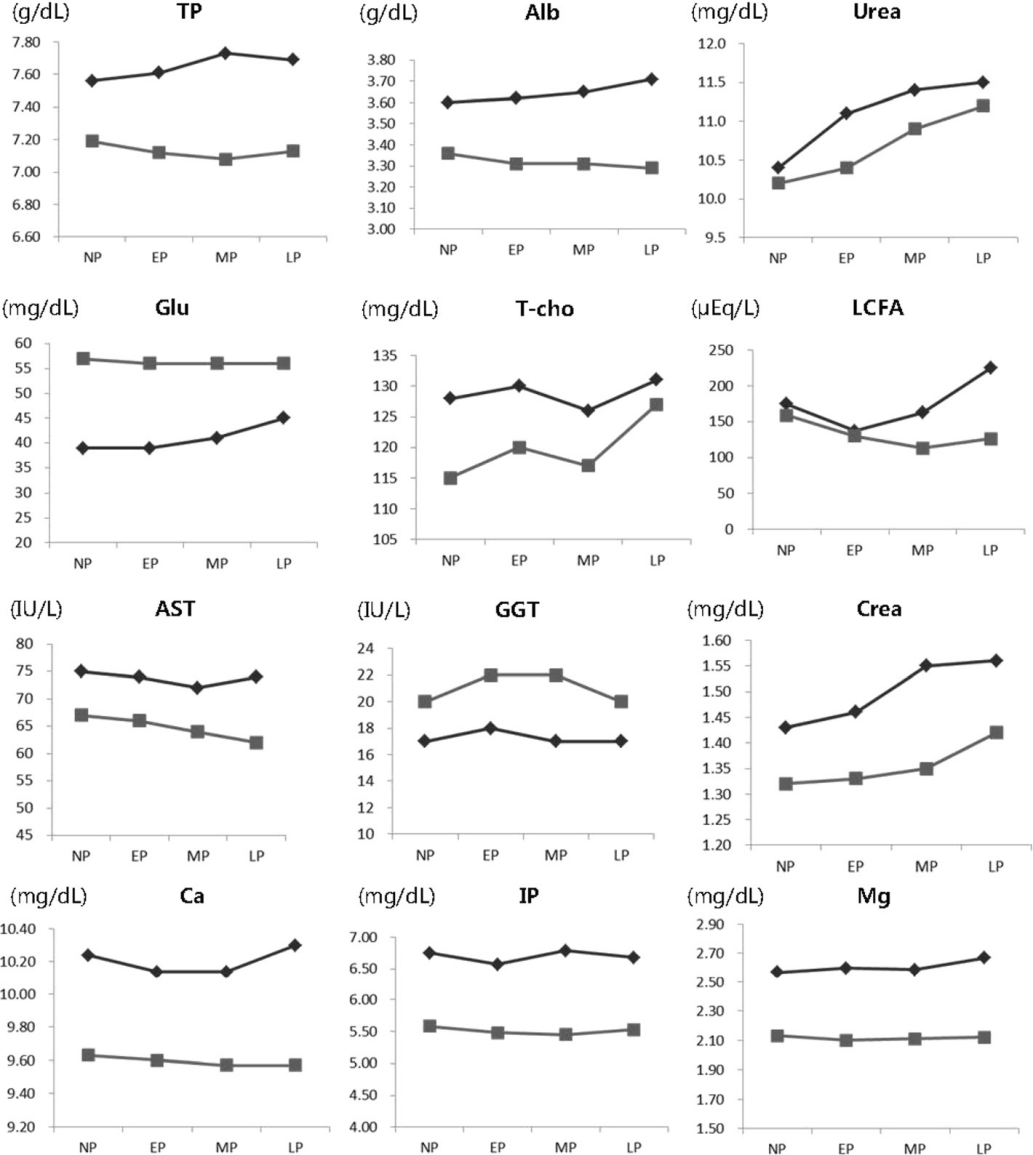
**Comparison of mean serum components between Korean Hanwoo cows (**  **) and Japanese Wagyu cows (**  **).** NP: non-pregnancy, EP: early pregnancy, MP: middle pregnancy, LP: late pregnancy.

The mean values of serum components in Korean Hanwoo and Japanese Wagyu cows were compared during four physiological stages (non-pregnant, early pregnancy, middle pregnancy and late pregnancy), because the physiology of these two breeds was known to be very similar. The results of this comparison, which are shown in Figure 1, revealed that the patterns of serum components were very similar in the two breeds. All the serum components were higher in Korean Hanwoo than in Japanese Wagyu cows during all test periods, with the exception of Glu and GGT. TP, Alb and UREA indicate the protein intake of an animal from diet [4]. In this experiment, mean values of these three indicators were higher in Korean Hanwoo than in Japanese Wagyu cows, in all physiological stages. This may indicate that the protein level in the diets of Korean Hanwoo cows was higher than in the diets of Japanese Wagyu cows. Blood Glu, T-cho and LCFA are the most commonly used blood metabolites to assess the energy metabolism. The physiological status of an animal affects the serum concentration of these related metabolites in energy metabolism. The elevated level of LCFA at MP and LP periods may indicate that there was a negative energy balance during pregnancy periods in Korean Hanwoo cows. On the other hand, the mean value of serum Glu was lower in Korean Hanwoo than in Japanese Wagyu cows. For this reason, we suggest that the elevated level of LCFA and the lowered level of Glu may be caused by an insufficient energy intake. Serum activity of AST and GGT indicate liver function, and Crea indicates kidney function. AST is an enzyme that expresses in many tissues, particularly in liver and cardiac muscle [13]. In this study, AST and Crea were higher in Korean Hanwoo than in Japanese Wagyu cows; however, GGT was lower in Korean Hanwoo than in Japanese Wagyu cows. Serum Ca, Mg and IP were higher in the Korean Hanwoo than in the Japanese Wagyu cows, indicating that the mineral level was higher in Korean Hanwoo cows’ diets than in Japanese Wagyu cows. In this study, the patterns of serum components were highly similar in the two breeds.

## Conclusions

In this study, the metabolic profile of the Korean Hanwoo cows was determined. The differences in the metabolic profile between Korean Hanwoo cows and Japanese Wagyu cows were also verified. Our findings may provide some basis for understanding the reproductive and feeding situations of Korean Hanwoo cows.
